# The automatic detection of diabetic kidney disease from retinal vascular parameters combined with clinical variables using artificial intelligence in type-2 diabetes patients

**DOI:** 10.1186/s12911-023-02343-9

**Published:** 2023-10-30

**Authors:** Shaomin Shi, Ling Gao, Juan Zhang, Baifang Zhang, Jing Xiao, Wan Xu, Yuan Tian, Lihua Ni, Xiaoyan Wu

**Affiliations:** 1 Department of Nephrology, Zhongnan Hospital of Wuhan University, 169 Donghu Road, Wuhan, 430071 Hubei China; 2 Xiangyang Central Hospital, Affiliated Hospital of Hubei University of Arts and Science, Xiangyang, Hubei, 441000 China; 3 Department of Biochemistry, Wuhan University TaiKang Medical School (School of Basic Medical Sciences), Wuhan, 430071 Hubei China; 4 Department of General Practice, Zhongnan Hospital of Wuhan University, 169 Donghu Road, Wuhan, 430071 Hubei China

**Keywords:** Diabetic kidney disease, Diabetic retinopathy, Type 2 diabetes, Fundus photography, Artificial intelligence

## Abstract

**Background:**

Diabetic kidney disease (DKD) has become the largest cause of end-stage kidney disease. Early and accurate detection of DKD is beneficial for patients. The present detection depends on the measurement of albuminuria or the estimated glomerular filtration rate, which is invasive and not optimal; therefore, new detection tools are urgently needed. Meanwhile, a close relationship between diabetic retinopathy and DKD has been reported; thus, we aimed to develop a novel detection algorithm for DKD using artificial intelligence technology based on retinal vascular parameters combined with several easily available clinical parameters in patients with type-2 diabetes.

**Methods:**

A total of 515 consecutive patients with type-2 diabetes mellitus from Xiangyang Central Hospital were included. Patients were stratified by DKD diagnosis and split randomly into either the training set (70%, *N* = 360) or the testing set (30%, *N* = 155) (random seed = 1). Data from the training set were used to develop the machine learning algorithm (MLA), while those from the testing set were used to validate the MLA. Model performances were evaluated.

**Results:**

The MLA using the random forest classifier presented optimal performance compared with other classifiers. When validated, the accuracy, sensitivity, specificity, F1 score, and AUC for the optimal model were 84.5%(95% CI 83.3–85.7), 84.5%(82.3–86.7), 84.5%(82.7–86.3), 0.845(0.831–0.859), and 0.914(0.903–0.925), respectively.

**Conclusions:**

A new machine learning algorithm for DKD diagnosis based on fundus images and 8 easily available clinical parameters was developed, which indicated that retinal vascular changes can assist in DKD screening and detection.

**Supplementary Information:**

The online version contains supplementary material available at 10.1186/s12911-023-02343-9.

## Introduction

Diabetic kidney disease (DKD) has already become the single largest cause of end-stage kidney disease (ESRD), and it was reported that even early stages of DKD presented an increased risk of cardiovascular disease [1]. Early detection of diabetic kidney disease will allow appropriate interventions and thus substantially reduce the health-care burden. Detection of diabetic kidney disease depends on measurement of albuminuria or estimated glomerular filtration rate (eGFR) [2], but increasing evidence has shown that some DKD patients will not develop albuminuria [3]. Moreover, the measurements of albuminuria or eGFR are not always convenient since serum or urine samples need to be obtained and they have limited precision at an earlier DKD stage [2]. Although promising new markers have been reported, none have presented better performance as a screening tool for DKD than albuminuria [4]. On the other hand, although kidney biopsy is an accurate approach to identify early disease, it is invasive and cannot be routinely used. Thus, there is a need for a noninvasive and easily available tool for diagnosing DKD.

Meanwhile, DKD and diabetic retinopathy (DR) are the most common microvascular complications of type 2-diabetes mellitus [5]. A close relationship between DR and DN has been reported in epidemiologic studies [6], since they share similar structural and physiological changes during early diabetes, which may be due to hyperglycemia, microangiopathy, inflammation, endothelial dysfunction, oxidative stress and other processes [7, 8]. As a result, DR measurements have the potential to allow optical monitoring of human microcirculation and to be a unique noninvasive predictor of DN [6]. In recent decades, a large number of studies have been conducted and have indicated that retinal changes measured from fundus photography are related to DKD, even without retinopathy [1, 9] However, the results were inconclusive [10], with limited sensitivity, specificity, convenience and extensibility.

In the past few years, machine learning techniques have been used for the diagnosis [11, 12], prediction, and prognostic analysis of DKD [13, 14]. Most of the diagnostic models developed for DKD are based on demographic information, biochemical parameters (such as triglycerides, serum uric acid, urea nitrogen, etc.), genetic information and so on [11, 12]. However, biochemical parameters depend on the collection of blood and urine samples, so the model does not have a prominent advantage over the current diagnosis of DKD (based on urinary microalbumin). On the other hand, two studies in the past year explored the diagnostic value of fundus images for chronic kidney disease (CKD) based on artificial intelligence deep learning technology. Sabanayagam et al. developed a diagnostic model for CKD using only fundus image information, but their diagnosis of CKD relied only on the estimated glomerular filtration rate (eGFR) [7], so the early diagnosis value is unclear. Kang Zhang et al. first used fundus photographic images to construct a diagnostic model for early DKD, and the area under the receiver operating characteristic curve (AUC) of the model was 0.800–0.864 [15]. This model directly input fundus images, but the defect lies in which specific image features are extracted; that is, the diagnostic principle is unknown.

Nowadays artificial intelligence has been increasingly used and has shown good performance in clinical diagnosis and treatment [7]. Thus, we aimed to develop a novel noninvasive detection algorithm for DKD using artificial intelligence machine learning technology based on measurable retinal vascular parameters from fundus photographs combined with several easily available clinical parameters in type-2 diabetes mellitus (T2DM) patients.

## Materials and methods

### Study design and population

Data from 528 sequential eligible inpatients with T2DM from Xiangyang Central Hospital from 4 January 2021 to 31 December 2021 were extracted retrospectively. Inclusion criteria were as follows: patients aged between 18–80 years old with T2DM who had UACR and eGFR data and clear retinal photographs. Full exclusion criteria were cataract or glaucoma (*n* = 1), severe systemic diseases (such as end-stage renal disease, severe heart or liver disease, or malignant tumor) or acute complications of DM (such as diabetic ketoacidosis, hyperglycemic hyperosmolar state, lactic acidosis, or hypoglycemia coma) (*n* = 3); and patients without gradable retinal photographs (*n* = 9). Finally, 515 patients were included.

The study protocol was approved by the Ethics Committee of Xiangyang Central Hospital, an affiliated hospital of Hubei University of Arts and Science (Ethics batch number: XYSZXYY-LLDD-PJ-2022–034). The study was registered at the Chinese Clinical Trial Center (Registration number: ChiCTR2200060132) and performed in accordance with the guidelines of the Declaration of Helsinki. Private personal information was removed during the process of analysis and publication. Informed consent exemptions were approved by the ethics committees.

### Definition of diabetic kidney disease

T2DM was defined as fasting plasma glucose ≥ 7.0 mmol/L, 2-h postprandial plasma glucose (2hPG) ≥ 11.1 mmol/L, glycated hemoglobin (HbA1c) ≥ 6.5%, symptoms plus random plasma glucose ≥ 11.1 mmol/L [16], self-reported physician diagnosed T2DM or use of antidiabetic medications. The urinary albumin to creatinine ratio (UACR) was calculated using spot urine collected in the morning. Albuminuria was categorized as normoalbuminuria (UACR < 30 mg/g creatinine) and albuminuria (UACR ≥ 30 mg/g creatinine). The estimated glomerular filtration rate (eGFR) was evaluated using the Chinese Chronic Kidney Disease Epidemiology Collaboration (CKD-EPI) formula as eGFR (ml/min/1.73 m^2^) = 175 × (serum creatinine mg/dL)^−1.154^ × (Age) ^−0.203^ (× 0.742 if female) [17]. UACR and serum creatinine were measured by a Beckman Coulter AU 680 (Brea, USA). DKD was defined as an eGFR of less than 60 ml/min/1.73 m^2^ or an albuminuria concentration of more than 30 mg/g after ruling out other possible causes of kidney injury [16]. If the data are available, only persistent albuminuria ≥ 30 mg/g, which is sustained over ≥ 90 days, will be considered “albuminuria”.

### Clinical risk factors

To train models to predict DKD, 8 demographic and clinical risk factors linked to diabetic kidney disease were used as predictors, based on previous studies [2, 7, 10], and they were extracted from the hospital’s electronic medical records system, including: age, gender, duration of diabetes, hypertension, history of cardiovascular and cerebrovascular disease, smoking, BMI (kg/m^2^), and glycosylated hemoglobin (%). Hypertension was defined as systolic blood pressure ≥ 140 mmHg or diastolic blood pressure ≥ 90 mmHg, self-reported physician-diagnosed hypertension or use of blood pressure-lowering medications. Cardiovascular and cerebrovascular diseases were defined as myocardial infarction, angina, heart failure or stroke. Body mass index (BMI) was calculated as follows: weight (kg) divided by height squared (m^2^). HbA1c was measured with finger capillary blood using test paper produced by Hangzhou Liwei Technology Co., Ltd., China.

### Fundus photographing

Color fundus photographs were obtained using a retinal fundus camera (Canon, CR-2, Japan) at 45° of both eyes for each patient. Mydriatic agents were not applied. Macula-centered images were collected. The left eye data were used, and when unavailable, the right eye data were used. The images were obtained in JPG format. For all subjects with more than one visit, the most recent data and images were used.

The measurement was performed using a mature automatic computer program developed by the Key Laboratory of Biomedical Information Engineering of Ministry of Education, School of Life Science and Technology, Xi’an Jiaotong University, China, which had been verified to be accurate and effective [1, 8, 18]. First, the deep learning algorithm was used to find the optic disc of the fundus and separate the arteries and veins. With the optic disc as the center, blood vessels were divided into three regions: central zone (0.5–1 times the diameter of optic disc [DD]), intermediate zone (1–2 times DD), and peripheral zone (> 2 times DD). The measured parameters included nonvascular area, total vessel tortuosity, total fractal dimension and vessel caliber. For vessel caliber, the above three regions were measured and averaged, and the arteries and veins were measured separately, but ultimately, the peripheral vein caliber and peripheral arterial caliber were selected as predictors since the difference in their measurements between DKD patients and non-DKD patients was most significant.

### Machine learning algorithm development

**Fig. 1:**
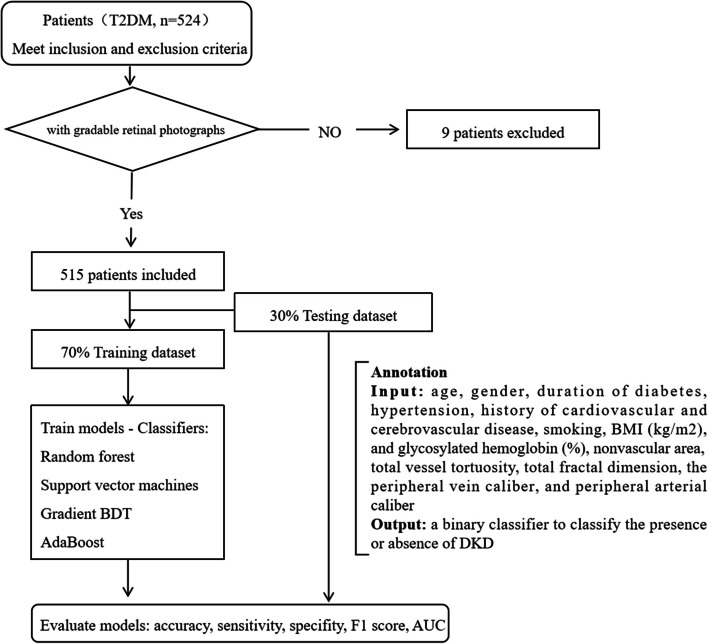
The workflow for developing machine learning models to detect diabetic kidney disease in this study

Python 3.6.13 (library, scikit-learn) was used to develop and validate the machine learning-based algorithm. Patients were stratified by DKD diagnosis and split randomly into either the training set (70%, *N* = 360) or the testing set (30%, *N* = 155) (random seed = 1). Data from the training set were used to develop the machine learning algorithm (MLA), while those from the testing set were used to validate the MLA. There was no overlap between the training set and the testing set. The input was measurable vascular parameters and 8 clinical parameters. The output was a binary classifier to classify the presence or absence of DKD. Four MLAs were developed using 4 different classifiers, including random forest (RF), support vector machine (SVM), gradient boosting decision tree (GBDT), and AdaBoost, to find an optimal detection model. Stratified tenfold cross-validation and grid search were used to search the optimal hyperparameters of classifiers to increase the performance of the models in the training cohort. In the RF model, Gini importance was used as a general measure of feature relevance. The accuracy, sensitivity, specificity, F1 score and area under the receiver operating characteristic curve (AUC) were calculated to evaluate the performance of the models. Predictive models were compared among classifiers, and the one with the best performance was selected (as summarized in Fig. 1).

### Statistical analysis

Python 3.6.13 (library, scikit-learn) was used for development and validation of the models. STATA Version 16 (Stata Corporation, College Station, TX, USA) was used for statistical analysis. Continuous variables are presented as the mean ± standard deviation, and categorical variables are presented as percentages. Student’s t test was used for continuous data, and the χ2 test was used for categorical data. The accuracy, sensitivity, specificity F1 score, and AUC were calculated to evaluate the performance of the prediction models.

A small number of missing data (2 patients were without duration of diabetes, 17 were without BMI, and 74 were without HbA1c) were imputed and replaced by the method of k-Nearest Neighbor (k-NN) imputation. In order to correct for dataset imbalance (149 patients with DKD in the overall cohort; 28.9%), two different oversampling algorithms were applied to all the models: random oversampling, and synthetic minority over-sampling technique (SMOTE). Overfitting bias was defined as the difference between the accuracy observed at training and accuracy at validation, and the learning curve was used to display the degree of fitting of the selected model. Statistical significance was indicated by a *P* value < 0.05 (two-sided).

## Results

### Basic characteristics and retinal measurements

The mean age of the 515 included patients was 54 years, and 329 (63.9%) were male. Moreover, 46.8% had hypertension, while 17.3% and 42.7% ever had cardiovascular/cerebrovascular diseases and smoking history, respectively. The median UACR and eGFR were 59.6 mg/g and 110.3 ml/min/1.73 m^2^, respectively.

Compared with T2DM patients without DKD, those with DKD were older, had a longer duration and higher BMI, and more frequently had hypertension or cardiovascular/cerebrovascular diseases and a smoking history (*P* < 0.05). Moreover, patients with DKD seemed to have a higher BMI, but the difference was not statistically significant (as shown in Table 1). Regarding the vascular parameters, patients with DKD had a higher nonvascular area and vessel tortuosity and a lower fractal dimension. Vessel calibers were increased, especially in the peripheral zone (*P* < 0.05).

**Table 1:** Baseline characteristics of participants

	non-DKD (*n* = 366)	DKD (*n* = 149)	Total (*n* = 515)	*P* value
**Risk factors**				
Age (year)	52.8 ± 0.51	57.3 ± 0.94	54.1 ± 0.46	< 0.05
Gender (Male %)	65.3	63.1	63.9	0.810
Duration (month)	71.7 ± 4.0	105.9 ± 7.0	81.6 ± 3.5	< 0.05
Hypertention	38.9	67.8	46.8	< 0.05
History (%)	13.9	26.2	17.3	< 0.05
Smoking (%)	42.5	45.0	42.7	0.799
BMI (kg/m^2^)	25.5 ± 0.19	26.0 ± 0.31	25.6 ± 0.16	0.109
HbA1c (%)	8.9 ± 0.13	9.7 ± 0.19	9.1 ± 0.12	< 0.05
UACR (mg/g)	11.8 ± 0.56	176.9 ± 20.91	59.6 ± 6.90	< 0.05
eGFR(ml/min/1.73m^2^)	115.2 ± 1.44	98.1 ± 3.53	110.3 ± 1.48	< 0.05
**Vascular parameters**				
NVArea /10^4^ (μm^2^)	163.24 ± 0.39	172.77 ± 0.97	166.00 ± 0.44	< 0.05
Tortuosity	0.481 ± 0.0005	0.483 ± 0.0009	0.481 ± 0.0108	0.0145
FD	1.4229 ± 0.0001	1.4105 ± 0.0218	1.4193 ± 0.0001	< 0.05
VW(All) (μm)				
Central	57.10 ± 0.25	57.64 ± 0.41	57.24 ± 0.20	0.2651
Middle	52.37 ± 0.24	53.57 ± 0.39	52.72 ± 0.20	0.0076
Peripheral	42.97 ± 0.18	45.13 ± 0.32	43.60 ± 0.16	< 0.05
VW(Vein) (μm)				
Central	57.18 ± 0.25	57.64 ± 0.41	57.32 ± 0.20	0.334
Middle	52.34 ± 0.23	53.56 ± 0.40	52.68 ± 0.20	0.0066
Peripheral	42.96 ± 0.18	45.15 ± 0.31	43.60 ± 0.16	< 0.05
VW(Artery) (μm)				
Central	42.97 ± 0.18	45.13 ± 0.32	48.24 ± 0.24	< 0.05
Middle	42.97 ± 0.18	45.13 ± 0.32	47.12 ± 0.20	< 0.05
Peripheral	42.83 ± 0.19	44.40 ± 0.33	43.28 ± 0.16	< 0.05

*Abbreviations: DKD* Indicates diabetic kidney disease, *BMI* Body mass index, *HbA1c* Glycosylated hemoglobin, *UACR* Urinary albuminuria creatinine ratio, eGFR Estimated glumerular filtration rate, *NVArea* Non-vascular area, *Tortuosity total* Vessel tortuosity, *FD total* Fractal dimension, *VW* Vessel width, *History* History of cardiovascular and cerebrovascular disease (myocardial infarction, angina, heart failure or stroke)

### Evaluations of the developed models for detecting diabetic kidney disease

After tuning of hyperparameters, a RF algorithm with SMOTE correction for data set imbalance was selected as the best model and was validated in the testing data set. A detailed description of the applied supervised learning methods is provided in Supplementary Table 1 and Supplementary Fig. 1, and the tunning of the RF model with SMOTE correction was shown in Supplementary Table 2.

**Fig. 2:**
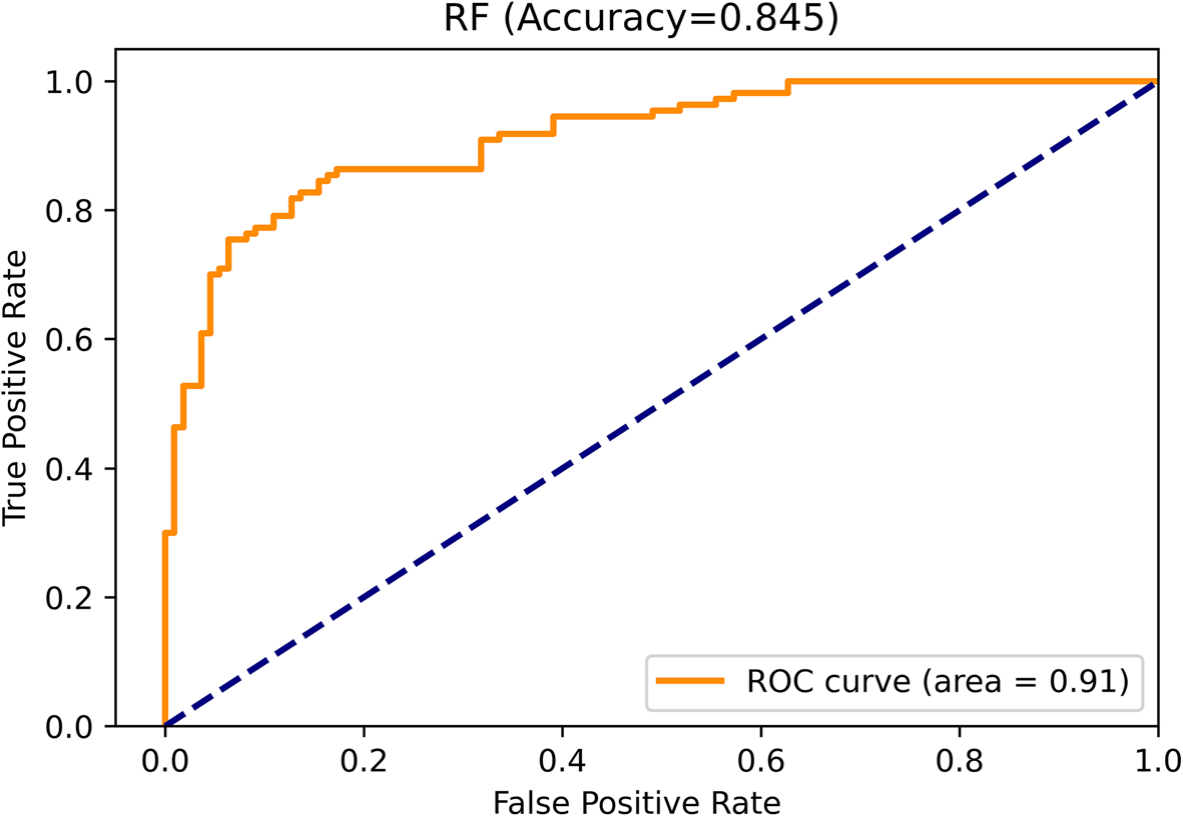
ROC curve of the best model using Random Forest classifier with SMOTE correction for data set imbalance in validation

**Fig. 3:**
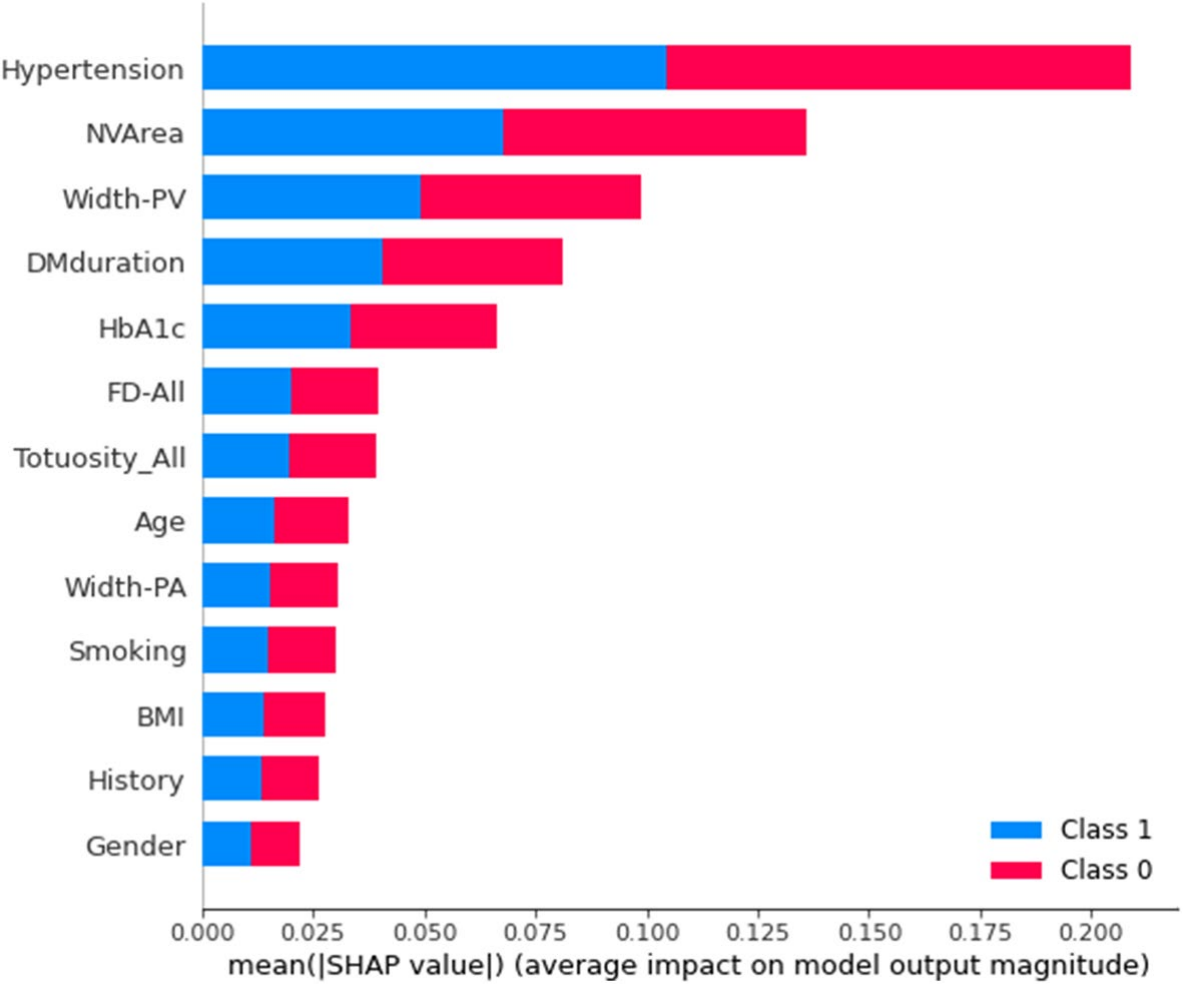
Relative variable importance for the accuracy of detecting diabetic kidney disease using the random Forest classifier with SMOTE correction for data set imbalance. Abbreviations: BMI indicates body mass index; HbA1c, glycosylated hemoglobin; NVArea, non-vascular area; Tor-All, total vessel tortuosity; FD-All, total fractal dimension; Width-PA, peripheral arterial width; Width-PV, the peripheral vein width; History, history of cardiovascular and cerebrovascular disease (myocardial infarction, angina, heart failure or stroke); SMOTE, synthetic minority over-sampling technique

It comprised 26 classification trees with a maximum number of 17 splits. One of the classification trees from this model using the RF classifier is presented in Supplementary Fig. 2. The accuracy, sensitivity, specificity, and F1 score for the optimal model in training were 90.0%(95% CI 89.5–90.5), 91.8%(91.0–92.6), 88.3%(87.7–88.9), and 0.902(0.896–0.908), respectively, and in validation were 84.5%(95% CI 83.3–85.7), 84.5%(82.3–86.7), 84.5%(82.7–86.3), and 0.845(0.831–0.859), respectively, with an AUC of 0.914(0.903–0.925) (ROC curve shown in Fig. 2). The optimal model had a good fit, with accuracies of 90.0% in training and 84.5% in validation (As shown in Supplemenrary Fig. 3). The importance of each variable to the optimal prediction model was analyzed by SHAP (Shapley Additive Explanations) (As shown in Fig. 3).

### Supplementary analyses

We further imputed the missing data by the method of backfilling missing values and retrained the machine learning models using the random forest model with SMOTE correction for data set imbalance, and the results were similar with an overall accuracy of 82.3%(95% CI 80.8–83.8) and AUC of 0.903(0.888–0.918) (as shown in Supplementary Fig. 4a). And when the missing data were replaced by the mean value, the results remained almost consistent with those before, with an overall accuracy of 83.2%(81.5–84.9) and AUC of 0.920(0.903–0.937) (as shown in Supplementary Fig. 4b). Moreover, a few tipical samples’ (including 3 DKD patients and 3 non-DKD patients) clinical indicators and fundus images were displayed in Supplementary Fig. 5.

## Discussion

DKD is the main cause of ESRD worldwide, accounting for approximately 20–40% of patients with diabetes [19, 20]. Early and accurate detection and intervention contributing to a better outcome is beneficial for the patients [13, 19]. The present detection of DKD depends on the measurement of albuminuria or the eGFR, which are invasive and not optimal. In the present study, we developed a new detection model for DKD based on retinal vascular parameters measured from fundus images and 8 easily available clinical parameters using artificial intelligence machine learning technology.

### Compared with previous studies

Early detection of DKD is of great importance to preventing the progression of nephropathy. Albuminuria or eGFR, currently used in clinical practice are not convenient enough and with limited precision at an earlier DKD stage [2]. Thus, multiple efforts have been made to explore new biomarkers for the early diagnosis of DKD. The potentical biomarkers of DKD studied in recent years included kidney injury molecule 1 (KIM-1), chemokines (such as CCL19, CCL5), multi-omics related biomarkers (such as CKD273 score, various miRNAs), and so on [21]. However, all have different limitations such as poor accuracy, sensitivity, reliability and convenience, which largely influence their clinial value, and none have presented better performance as a screening tool for DKD than albuminuria [4]. Although kidney biopsy is accurate as the gold standard for the diagnosis of DKD, it is invasive and cannot be routinely used. Thus, noninvasive and easily available tools for detecting DKD are needed.

AI machine learning based on traditional risk factors has been increasingly used for the prediction of diabetic complications [22], chronic kidney disease, acute kidney injury, kidney function decline and so on, with an accuracy of 76–85.2% [13, 23–27]. For example, Makino developed a prediction model for 6-month DKD aggravation in patients with DM with 71% accuracy [20], and Belur Nagaraj et al. developed a prediction model for long-term ESRD in subjects with T2DM with 18 demographic and clinical parameters [23]. Few studies have focused on the detection of DKD. Maniruzzaman et al. developed a model with superior performance to detect DN using SVM-RBF (support vector machine-radial basis function) with 13 parameters (sex, age, BMI, DM duration, FBS, HbA1c, LDL, HDL, TGs, SBP, DBP, DM treatment, use of statins), and the accuracy and AUC were 88.7% and 0.9, respectively, when validated [22]. Satish Kumar David et al. developed a detection model for DKD with an accuracy of 93.7% using IBK and random tree classification techniques through 18 parameters (age, sex, serum albumin, sodium, potassium, urea, glucose, creatinine, HbA1c, hemoglobin, white blood cell counts, red blood cell counts, hemoglobin (%), platelet counts, SBP, DBP, hypertension, and retinopathy) [19]. However, most biochemical parameters are not convenient enough, and retinal vascular parameters have not been used in formal models, although a large number of studies have indicated that retinal changes are related to DKD [1, 9].

On the other hand, AI deep learning, which uses imaging data as a subset of machine learning, has achieved many recent advancements in the detection of various diseases over the past 20 years, including medical kidney diseases [28]. To the best of our knowledge, only four relevant studies are available. Chin-Chi Kuo et al. developed a prediction model for CKD (GFR of < 60 ml/min/1.73 m2) with 4505 ultrasound-based images of the kidney, with an accuracy of 85.6% and AUC of 0.904 without external validation [29]. Kitamura et al. developed a diagnostic algorithm for DKD based on pathological immunofluorescent images of kidneys, with an accuracy of 83.28 ± 11.64% [30]. Sabanayagam et al. developed a diagnostic model for CKD using only fundus image information, but their diagnosis of CKD relied only on the estimated glomerular filtration rate (eGFR) [7], so the early diagnosis value is unclear. Kang Zhang et al. first used fundus photographic images to construct a diagnostic model for early DKD, and the area under the receiver operating characteristic curve (AUC) of the model was 0.800–0.864 [15]. This model directly input fundus images, but the defect lies in which specific image features are extracted; that is, the diagnostic principle is unknown.

Our model was the first to use retinal vascular parameters and 8 easily available clinical parameters combined by machine learning to detect DKD [16], with an overall accuracy of 84.5%.

### The strength of the present study

Although the performance of our model is not as good, it was the first to integrate data of measurable retinal vascular parameters and simple risk factor information to detect DKD, which is a noninvasive, low-cost, nonradioactive and easily available option and can be widely used as a real-time screening and diagnostic tool, since DR is suggested to be screened routinely by fundus photography annually in diabetes care [31]. Although the cost of the measurement of albuminuria or eGFR, the present detection methods of DKD, is already low, and we have not performed cost-effectiveness studies, the present model may reduce the screening cost further, since it may be integrated into the fundus cameras in the future which can screen for DKD while screening for DR at no additional cost. It has the potential to minimize unnecessary detection procedures for DKD, especially in primary care institutions with limited resources, thus improving the efficiency of the current medical system. Moreover, our model captured some information from the entire retinal images that cannot be recognized by doctors prior to clinical DKD diagnosis, such as changes in peripheral vascular caliber, vessel rarefaction and other changes; therefore, it is possible that it has some early diagnostic value beyond albuminuria or at least assists the present diagnosis method, which deserve further study. Thus, the present model sheds light on the future development of a better detection method for DKD.

### Analysis of the shortcomings and limitations

There are some limitations in this study. First, this is a retrospective study. The dataset used for the present model had a specific demographic sample, so we cannot guarantee its performance in other populations. Second**,** the study is cross-sectional, limiting the ability to track changes in retinal parameters or clinical variables over time, and the output of our study is binary classifier to classify the presence or absence of DKD, instead of a spectrum. Third, a sample size of 515 patients may be insufficient to capture the significant heterogeneity of DKD. Fourth, DKD may be closely associated with other factors not incorporated into the analysis, such as medication use, family history, lifestyle factors and so on. Fifth, in the present study, DKD was defined as an eGFR of less than 60 ml/min/1.73 m^2^ or an albuminuria concentration of more than 30 mg/g and not based on pathology, and whether albuminuria is persistent (sustained over ≥ 90 days) could only be confirmed in a few patients. Sixth, the present model lacks prospective validation and external validation. Seventh, we were unable to elucidate the interpretability of the present model, which is considered as a common "black box" property of machine learning algorithms. Based on the above considerations, it is possible to optimize the model in the future. In addition, how will the present model to be fitted into the existing clinical workflows and its efficacy to the existing standard tests need to be clarified in the future.

On the other hand, the performance of the model was not good enough, which may be determined by the differences between the pathogenesis of DKD and that of DR, since they were not always consistent. Although DR and DN share similar structural and physiological changes during early diabetes [7, 8], their causes are not absolutely the same, as they have different pathogenic cytokines and different associations with neuropathy, obesity, hypertension and so on [6, 9, 32]. Thus, better studies with larger samples and studies that are based on pathological diagnoses are needed.

## Conclusion

In conclusion, a new machine learning algorithm for DKD diagnosis based on fundus images and 8 easily available clinical parameters was developed, and it shed light on areas for future exploration, which indicated that fundus photography can be used as an adjunctive tool for screening and detecting DKD.

### Supplementary Information

**Additional file 1:** **Supplementary Figure 1.** The ROC curves of machine learning models in validation and data imbalance correction.

**Additional file 2:** **Supplementary Figure 2.** One of the classification trees in the model using the Random Forest classifier for the detecting of DKD.

**Additional file 3:** **Supplementary Figure 3.** The learning curve of the model to detect diabetic kidney disease using Random Forest classifier with SMOTE correction.

**Additional file 4:** **Supplementary Figure 4.** ROC curve of the best model using Random Forest classifier with SMOTE correction for data set imbalance in validation. a: missing data imputed by the method of backfilling missing values; b: missing data replaced by the mean value.

**Additional file 5:** **Supplementary Figure 5.** A few tipical samples’ (including 3 DKD patients and 3 non-DKD patients) clinical indicators.

**Additional file 6:** **Supplementary Table 1.** The accuracy of machine learning models and data imbalance correction.

**Additional file 7:** **Supplementary Table 2.** Tunning of the machine learning model.

**Additional file 8.** Original data.

**Additional file 9.** Supplementary Python source codes. Code1: Python code for the model using RF classifier with SMOTE correction for data set imbalance. Code2: Python code for the model using SVM classifier with SMOTE correction for data set imbalance. Code3: Python code for the model using BDT classifier with SMOTE correction for data set imbalance. Code4: Python code for the model using Ada classifier with SMOTE correction for data set imbalance. Code5: Python code for the model using RF classifier with Random oversampling correction for data set imbalance. Code6: Python code for the model using SVM classifier with Random oversampling correction for data set imbalance. Code7: Python code for the model using BDT classifier with Random oversampling correction for data set imbalance. Code8: Python code for the model using Ada classifier with Random oversampling correction for data set imbalance. Code9: Python code for the model using RF classifier with no correction for data set imbalance. Code10: Python code for the model using SVM classifier with no correction for data set imbalance. Code11: Python code for the model using BDT classifier with no correction for data set imbalance. Code12: Python code for the model using Ada classifier with no correction for data set imbalance. Code13: Python code for the ROC curves of models with SMOTE correction for data set imbalance. Code14: Python code for the ROC curves of models with Random oversampling correction for data set imbalance. Code15: Python code for the ROC curves of models with no correction for data set imbalance. Code16: Python code for the model using RF classifier with SMOTE correction for data set imbalance, and imputing the missing data by the method of backfilling missing values. Code17: Python code for the model using RF classifier with SMOTE correction for data set imbalance, and imputing the missing data by means. Code18: Python code for tunning of the model using RF classifier with SMOTE correction for data set imbalance. Code19: Python code for calculating the standard deviations.

## Data Availability

The data in this study was extracted retrospectively from electronic medical record system of Xiangyang Central Hospital from 4 January 2021 to 31 December 2021. The datasets are not publicly available, but are available on request from the corresponding author on reasonable request.
