# Supplementary material for: The automatic detection of diabetic kidney disease from retinal vascular parameters combined with clinical variables using artificial intelligence in type-2 diabetes patients
Source: BMC Med Inform Decis Mak. 2023 Oct 30;23:241. doi: 10.1186/s12911-023-02343-9 (PMC10617171; doi:10.1186/s12911-023-02343-9)
Supplement: Supplementary file 1 — Additional file 1: Supplementary Figure 1. The ROC curves of machine learning models in validation and data imbalance correction. [file 12911_2023_2343_MOESM1_ESM.doc]

**Supplementary Figure 1** The ROC curves of machine learning models in validation and data imbalance correction


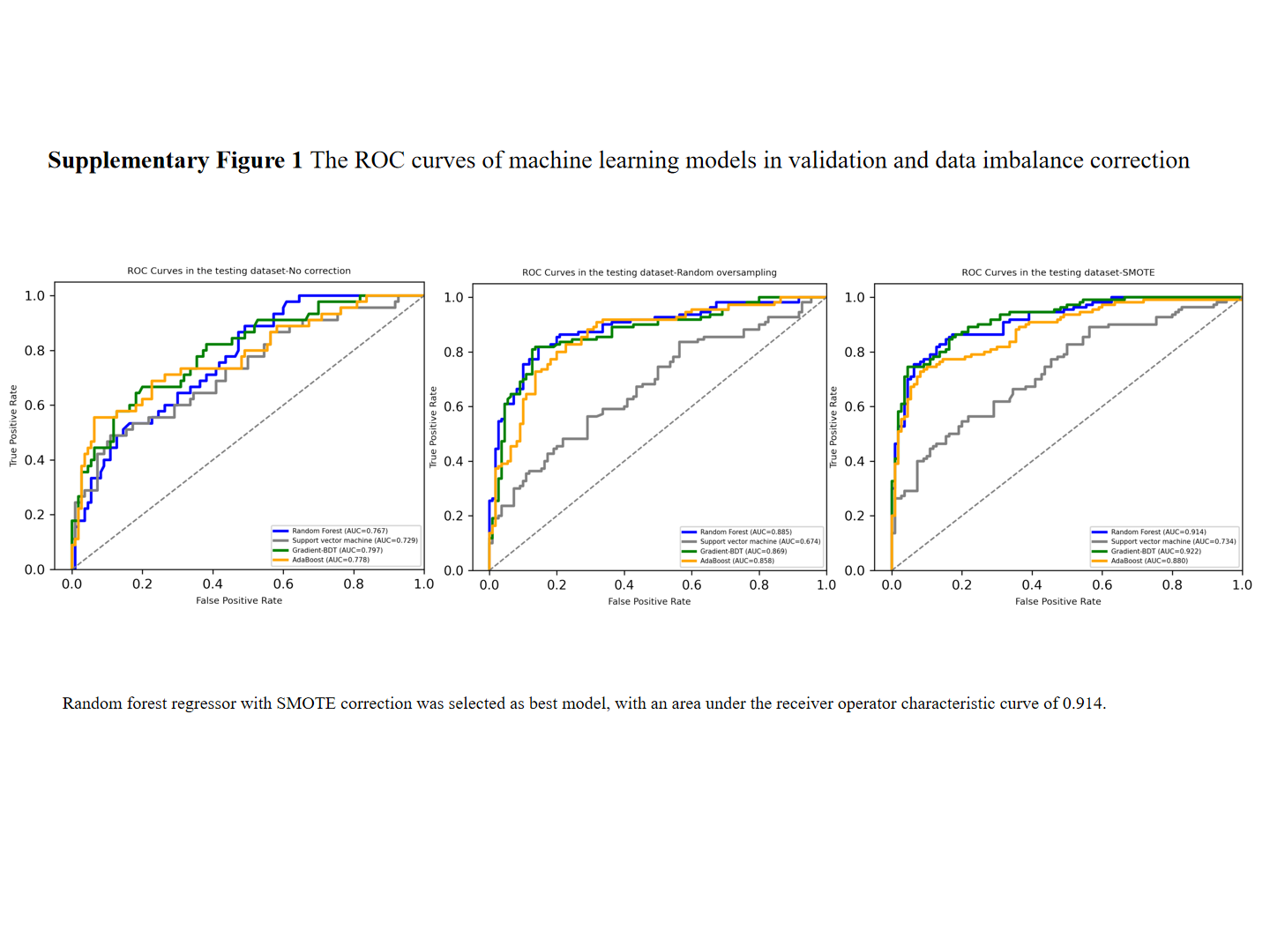


Random forest regressor with SMOTE correction was selected as best model, with an area under the receiver operator characteristic curve of 0.914.
