# Supplementary material for: The automatic detection of diabetic kidney disease from retinal vascular parameters combined with clinical variables using artificial intelligence in type-2 diabetes patients
Source: BMC Med Inform Decis Mak. 2023 Oct 30;23:241. doi: 10.1186/s12911-023-02343-9 (PMC10617171; doi:10.1186/s12911-023-02343-9)
Supplement: Supplementary file 2 — Additional file 2: Supplementary Figure 2. One of the classification trees in the model using the Random Forest classifier for the detecting of DKD. [file 12911_2023_2343_MOESM2_ESM.doc]

**Supplementary Figure 2** One of the classification trees in the model using the Random Forest classifier for the detecting of DKD





It comprised 26 classification trees with a maximum number of 17 splits. One of the classification trees from this model using the RF classifier is presented here.
