# Supplementary material for: The automatic detection of diabetic kidney disease from retinal vascular parameters combined with clinical variables using artificial intelligence in type-2 diabetes patients
Source: BMC Med Inform Decis Mak. 2023 Oct 30;23:241. doi: 10.1186/s12911-023-02343-9 (PMC10617171; doi:10.1186/s12911-023-02343-9)
Supplement: Supplementary file 3 — Additional file 3: Supplementary Figure 3. The learning curve of the model to detect diabetic kidney disease using Random Forest classifier with SMOTE correction. [file 12911_2023_2343_MOESM3_ESM.doc]

**Supplementary Figure 3** The learning curve of the model to detect diabetic kidney disease using Random Forest classifier with SMOTE correction


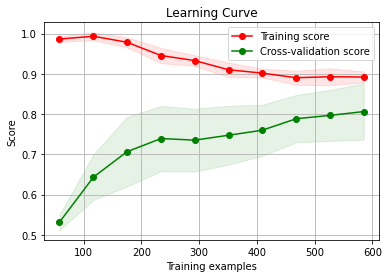


The optimal model using Random Forest classifier with SMOTE correction, had a good fit, with accuracies of 90.0% in training and 84.5% in validation.
