# Supplementary material for: The automatic detection of diabetic kidney disease from retinal vascular parameters combined with clinical variables using artificial intelligence in type-2 diabetes patients
Source: BMC Med Inform Decis Mak. 2023 Oct 30;23:241. doi: 10.1186/s12911-023-02343-9 (PMC10617171; doi:10.1186/s12911-023-02343-9)
Supplement: Supplementary file 4 — Additional file 4: Supplementary Figure 4. ROC curve of the best model using Random Forest classifier with SMOTE correction for data set imbalance in validation. a: missing data imputed by the method of backfilling missing values; b: missing data replaced by the mean value. [file 12911_2023_2343_MOESM4_ESM.doc]

**Supplementary Figure 4** ROC curve of the best model using Random Forest classifier with SMOTE correction for data set imbalance in validation. **a:** missing data imputed by the method of backfilling missing values; **b:** missing data replaced by the mean value


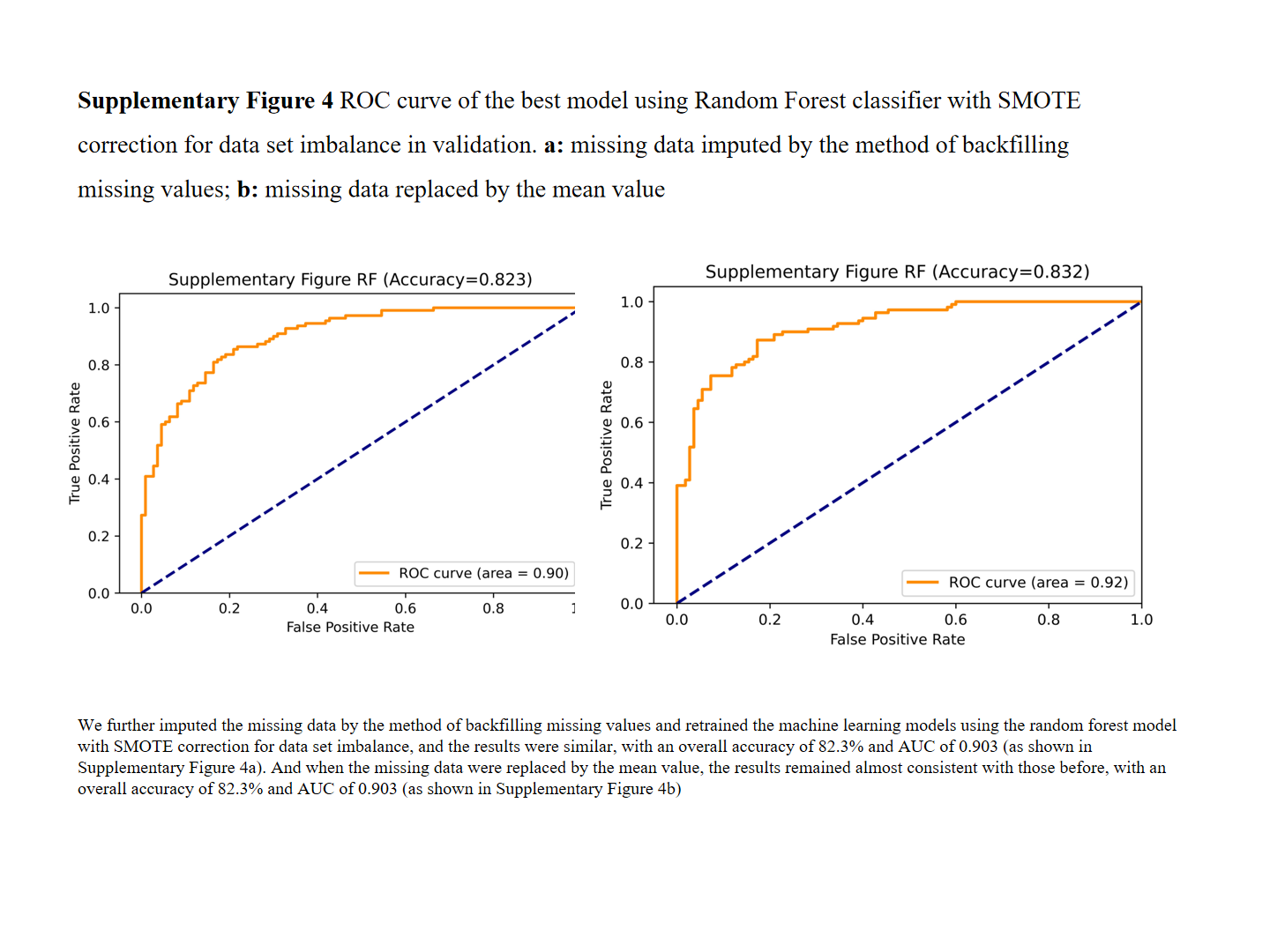


We further imputed the missing data by the method of backfilling missing values and retrained the machine learning models using the random forest model with SMOTE correction for data set imbalance, and the results were similar, with an overall accuracy of 82.3% and AUC of 0.903 (as shown in **Supplementary Figure 4a**). And when the missing data were replaced by the mean value, the results remained almost consistent with those before, with an overall accuracy of 83.2% and AUC of 0.920 (as shown in **Supplementary Figure 4b**)
