# Supplementary material for: The automatic detection of diabetic kidney disease from retinal vascular parameters combined with clinical variables using artificial intelligence in type-2 diabetes patients
Source: BMC Med Inform Decis Mak. 2023 Oct 30;23:241. doi: 10.1186/s12911-023-02343-9 (PMC10617171; doi:10.1186/s12911-023-02343-9)
Supplement: Supplementary file 5 — Additional file 5: Supplementary Figure 5. A few tipical samples’ (including 3 DKD patients and 3 non-DKD patients) clinical indicators. [file 12911_2023_2343_MOESM5_ESM.doc]

**Supplementary Figure 5** A few tipical samples’ (including 3 DKD patients and 3 non-DKD patients) clinical indicators


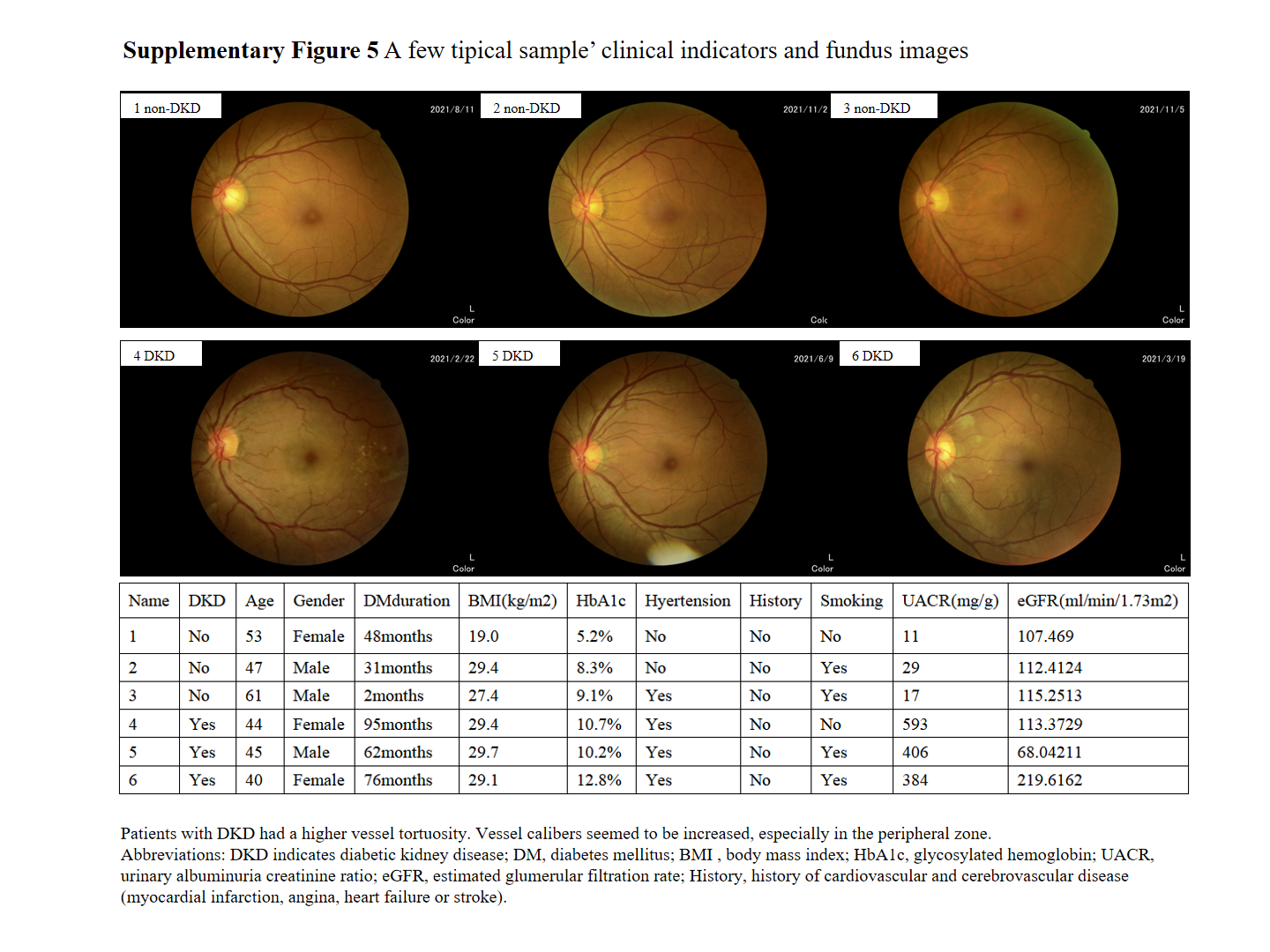


| Name | DKD | Age | Gender | DMduration | BMI(kg/m2) | HbA1c | Hyertension | History | Smoking | UACR(mg/g) | eGFR(ml/min/1.73m2) |
| --- | --- | --- | --- | --- | --- | --- | --- | --- | --- | --- | --- |
| 1 | No | 53 | Female | 48months | 19.0 | 5.2% | No | No | No | 11 | 107.469 |
| 2 | No | 47 | Male | 31months | 29.4 | 8.3% | No | No | Yes | 29 | 112.4124 |
| 3 | No | 61 | Male | 2months | 27.4 | 9.1% | Yes | No | Yes | 17 | 115.2513 |
| 4 | Yes | 44 | Female | 95months | 29.4 | 10.7% | Yes | No | No | 593 | 113.3729 |
| 5 | Yes | 45 | Male | 62months | 29.7 | 10.2% | Yes | No | Yes | 406 | 68.04211 |
| 6 | Yes | 40 | Female | 76months | 29.1 | 12.8% | Yes | No | Yes | 384 | 219.6162 |

Patients with DKD had a higher vessel tortuosity. Vessel calibers seemed to be increased, especially in the peripheral zone.

Abbreviations: DKD indicates diabetic kidney disease; DM, diabetes mellitus; BMI , body mass index; HbA1c, glycosylated hemoglobin; UACR, urinary albuminuria creatinine ratio; eGFR, estimated glumerular filtration rate;

History, history of cardiovascular and cerebrovascular disease (myocardial infarction, angina, heart failure or stroke).
