# Supplementary material for: The automatic detection of diabetic kidney disease from retinal vascular parameters combined with clinical variables using artificial intelligence in type-2 diabetes patients
Source: BMC Med Inform Decis Mak. 2023 Oct 30;23:241. doi: 10.1186/s12911-023-02343-9 (PMC10617171; doi:10.1186/s12911-023-02343-9)
Supplement: Supplementary file 6 — Additional file 6: Supplementary Table 1. The accuracy of machine learning models and data imbalance correction. [file 12911_2023_2343_MOESM6_ESM.doc]

**Supplementary Table 1** The accuracy of machine learning models and data imbalance correction

| DKD diagnosis  Training and testing | Model 1  Accuracy(%) | Model 2  Accuracy(%) | Model 3  Accuracy(%) | Model 4  Accuracy(%) |
| --- | --- | --- | --- | --- |
| No correction for data imbalance | 81.4  [72.9] | 71.7  [71.0] | 86.1  [78.7] | 87.8  [78.1] |
| Random oversampling | 95.8  [80.9] | 83.9  [75.9] | 88.5  [81.8] | 88.9  [78.6] |
| SMOTE | **90.0**  **[84.5]** | 86.9  [77.7] | 89.1  [83.6] | 89.6  [80.9] |

Accuracy is reported at training (above) and after 10-fold cross validation (below, square brackets) for 4 different machine learning (ML) models, with or without 2 algorithms for dataset imbalance correction. *Model 1 – Random Forest regressor; Model 2 –Support Vector Machines; Model 3 – Gradient Boosting Decision Tree; Model 4 – Adaboost; Random forest regressor with SMOTE correction was selected as best model and shown in bold. SMOTE indicates Synthetic Minority Oversampling Technique.
