# Supplementary material for: The automatic detection of diabetic kidney disease from retinal vascular parameters combined with clinical variables using artificial intelligence in type-2 diabetes patients
Source: BMC Med Inform Decis Mak. 2023 Oct 30;23:241. doi: 10.1186/s12911-023-02343-9 (PMC10617171; doi:10.1186/s12911-023-02343-9)
Supplement: Supplementary file 7 — Additional file 7: Supplementary Table 2. Tunning of the machine learning model. [file 12911_2023_2343_MOESM7_ESM.doc]

**Supplementary Table 2** Tunning of the machine learning model

|  | Tree(n) | MaxLeaf(n) | Accuracy(%) | Sensitivity(%) | Specifity(%) |
| --- | --- | --- | --- | --- | --- |
| Random Forest+SMOTE | 5 | 6 | 79.1 | 79.1 | 79.1 |
|  | 5 | 12 | 79.5 | 77.3 | 81.8 |
|  | 5 | 18 | 81.8 | 80.9 | 82.7 |
|  | 5 | 24 | 85.0 | 85.5 | 85.5 |
|  | 5 | 30 | 84.1 | 85.5 | 82.7 |
|  | 10 | 6 | 80.9 | 82.7 | 79.1 |
|  | 10 | 12 | 81.4 | 81.8 | 80.9 |
|  | 10 | 18 | 82.7 | 84.5 | 80.9 |
|  | 10 | 24 | 84.9 | 86.4 | 81.8 |
|  | 10 | 30 | 84.1 | 85.5 | 82.7 |
|  | 20 | 6 | 79.1 | 79.1 | 79.1 |
|  | 20 | 12 | 81.4 | 81.8 | 80.9 |
|  | 20 | 18 | 84.5 | 84.5 | 84.5 |
|  | 20 | 24 | 84.1 | 83.6 | 84.5 |
|  | 20 | 30 | 84.1 | 83.6 | 84.5 |
|  | 26 | 6 | 78.6 | 75.5 | 81.8 |
|  | 26 | 12 | 82.3 | 81.8 | 82.7 |
|  | **26** | **17** | **84.5** | **84.5** | **84.5** |
|  | 26 | 24 | 83.6 | 83.6 | 83.6 |
|  | 26 | 30 | 84.1 | 83.6 | 84.5 |

Hyperparameters (number of classification trees and maximum number of leaves) were tuned for the selected random forest regressor model to obtain the maximum accuracy in the training cohort. Sensitivity, specificity, and accuracy were assessed by 10-fold cross validation algorithm. The best model was a random forest composed by 26 classification trees with a maximum number of leaves equal to 17 (shown in bold). SMOTE indicates synthetic minority over-sampling technique.
